# Genomic reconstruction and features of glycosylation pathways in the apicomplexan *Cryptosporidium* parasites

**DOI:** 10.3389/fmolb.2022.1051072

**Published:** 2022-11-17

**Authors:** Dongqiang Wang, Chenchen Wang, Guan Zhu

**Affiliations:** Key Laboratory of Zoonosis Research of the Ministry of Education, Institute of Zoonosis, College of Veterinary Medicine, Jilin University, Changchun, China

**Keywords:** cryptosporidium, glycosylation pathway, N-glycans, O-glycans, GPI anchor biosynthesis

## Abstract

*Cryptosporidium* is a genus of apicomplexan parasites infecting humans or other vertebrates. The majority of the *Cryptosporidium* species live in host intestines (e.g., *C. parvum*, *C. hominis* and *C. ubiquitum*), but there are a few gastric species (e.g., *C. muris* and *C. andersoni*). Among them, *C. parvum* is the most important zoonotic species, for which a number of glycoproteins have been reported for being involved in the interacting with host cells. However, little is known on the cryptosporidium glycobiology. Information on the glycosylation pathways in *Cryptosporidium* parasites remains sketchy and only a few studies have truly determined the glycoforms in the parasites. Here we reanalyzed the *Cryptosporidium* genomes and reconstructed the glycosylation pathways, including the synthesis of *N*- and *O*-linked glycans and GPI-anchors. In *N*-glycosylation, intestinal *Cryptosporidium* possesses enzymes to make a simple precursor with two terminal glucoses on the long arm (i.e., Glc_2_Man_5_GlcNAc_2_ vs. Glc_3_Man_9_GlcNAc_2_ in humans), but gastric species only makes a simpler precursor containing only the “core” structure (i.e., Man_3_GlcNAc_2_). There is an ortholog of glucosidase II (GANAB) in all *Cryptosporidium* species, for which the authenticity is questioned because it contains no signal peptide and exist in gastric species lacking terminal glucoses for the enzyme to act on. In *O*-linked glycosylation, all *Cryptosporidium* species may attach one-unit HexNAc (GalNAc and GlcNAc) and two-unit Fuc-type (Man-Fuc) glycans to the target proteins. *Cryptosporidium* lacks enzymes to further process *N*- and *O*-glycans in the Golgi. The glycosylphosphatidylinositol (GPI)-anchor in *Cryptosporidium* is predicted to be unbranched and unprocessed further in the Golgi. *Cryptosporidium* can synthesize limited nucleotide sugars, but possesses at least 12 transporters to scavenge nucleotide sugars or transport them across the ER/Golgi membranes. Overall, *Cryptosporidium* makes much simpler glycans than the hosts, and the *N-*glycoforms further differ between intestinal and gastric species. The *Cryptosporidium N*- and *O*-glycans are neutrally charged and have limited capacity to absorb water molecules in comparison to the host intestinal mucins that are negatively charged and highly expandable in waters.

## Introduction

*Cryptosporidium* is a genus of protozoan parasites under the Phylum Apicomplexa that contains many pathogens of medical and veterinary importance (e.g., *Toxoplasma, Cyclospora, Eimeria, Plasmodium, Babesia* and *Theileria*). Among more than 40 known *Cryptosporidium* species, *C. parvum* is the most important zoonotic pathogen infecting humans and many other mammals including domesticated bovids (Checkley et al., 2015; Ryan et al., 2016; Pumipuntu and Piratae, 2018; Innes et al., 2020). Humans and animals with weakened or compromised immunity are more vulnerable to severe to deadly cryptosporidial infection. As a member of Apicomplexa, *Cryptosporidium* possesses an apical complex comprised of a set of unique apical cytoskeletal structures (e.g., apical rings, conoid and microtubes) and secretory organelles (i.e., rhoptries, micronemes and dense granules). Although the morphology and lifecycle of *Cryptosporidium* assemble intestinal coccidia (e.g., *Eimeria*, *Cyclospora* and *Isospora*), *Cryptosporidium* species are in fact highly divergent from the coccidia and other apicomplexans. Evolutionarily, they form a single phylogenetic clade at the base of the Apicomplexa, rather than a sister to the coccidia (Zhu et al., 2000a; Madern et al., 2004). Morphologically, they lack an apicoplast and a classic mitochondrion as well as their organellar genomes, but possess two unique central microtubular filaments (Zhu et al., 2000b; Templeton et al., 2010; Wang et al., 2021). Metabolically, *Cryptosporidium* lacks *de novo* synthetic pathways for any nutrients (e.g., nucleosides, amino acids, fatty acids and isoprenoids) (Abrahamsen et al., 2004; Xu et al., 2004; Rider and Zhu, 2010; Baptista et al., 2022).

Protein glycosylation, including the attachment of glycans to the asparagine (i.e., *N*-linked glycosylation) or the serine/threonine (i.e., *O*-linked glycosylation) residues and that of glycosylphosphatidylinositol (GPI)-anchor to the C-terminus of a protein, is one of the major post-translational modifications (Cherepanova et al., 2016; Kinoshita and Fujita, 2016; Corfield, 2017; Reily et al., 2019; Kinoshita, 2020; Toustou et al., 2022). Most glycosylated proteins (glycoproteins) are secretory, being distributed to the cell surface as transmembrane proteins or discharged as extracellular proteins. In the hosts (humans and animals), glycoproteins are known to be involved in every biological process in or around the cells, such as functioning in the structure, reproduction, immune system, hormones, intercellular communications and protection of cells and organisms (Moremen et al., 2012; Varki, 2017; Schjoldager et al., 2020). In the parasites in general, glycosylated and GPI-anchored proteins are more noticeable for their roles in the host-parasite interaction, including parasite adhesion/attachment to host cells and stimulation/evasion of the host immune responses (Anantharaman et al., 2007; Paschinger and Wilson, 2019; Lin et al., 2020; Mule et al., 2020).

In *Cryptosporidium*, a number of glycoproteins have been identified as, or predicted to be, glycosylated (e.g., gp900, gp15, gp40 and a number of mucin-like glycoproteins) (Wanyiri and Ward, 2006; Lendner and Daugschies, 2014). The presence of glycans in the immunodominant antigen gp900 was confirmed earlier by deglycosylation with *N*-glycosidase F (Petersen et al., 1992). Mass spectrometry-based studies of proteins from *C. parvum* oocysts have detected the presence of *O*-linked glycans in four proteins (i.e., gp900, gp40, gp15 and gp20) and *N*-linked glycans in 16 proteins (e.g., gp900, POWP1, GOP50 and seven other unique *Cryptosporidium* glycoproteins) (Haserick et al., 2017a; Haserick et al., 2017b). Among them, gp40 and gp15 are derived from precursor gp60 by the cleavage of furin-like protease. The C-segment gp15 (aka Cp17 in some earlier studies) and a low molecular weight antigen were found to be attached to the membrane by GPI-anchor (Priest et al., 2001; Priest et al., 2003). These studies implied the importance of protein glycosylation in the parasite based on the roles of these proteins in interacting with host cells during the parasite invasion and/or in serving as immunodominant antigens (Wanyiri and Ward, 2006; Lendner and Daugschies, 2014). Some vaccine candidates for cryptosporidiosis are also glycoproteins (Ifeonu et al., 2016; Haserick et al., 2017a). Glycosylation at the binding sites affects the protein-ligand interactions, including those between two proteins, proteins and small molecules, as well as antigen/epitopes and antibodies. Therefore, understanding the glycoforms in *Cryptosporidium* is important in the study of molecular interactions during the parasite invasion and development, host immunological responses and vaccine development.

The *C. parvum* genomes encode around 4,000 proteins, or 3,994 proteins to be exact for the Iowa-II reference strain, based on the annotated at the CryptoDB (https://www.CryptoDB.org) (Abrahamsen et al., 2004; Xu et al., 2004; Amos et al., 2022). These include some known glycoproteins and a large number of candidate proteins for glycosylation. Our reanalysis of the annotated proteins with Phobius have identified over one-third of them contain N-terminal signal peptide (SP) and/or transmembrane domains (TMDs) for targeting to the endoplasmic reticulum (ER). Around 800 proteins contain SP but lack TMD, or as type I (one TMD and SP) or type II/III (one TMD but lacking SP) transmembrane proteins that are candidates of extracellular proteins with high probability for glycosylation. Enzymes for glycosylation can also be identified in the annotated genomes for potentially synthesizing *O*- and *N*-glycans and the GPI-anchors. Mass spectrometry-based assays also showed that the glycans in *C. parvum* were much simpler than those in humans and animals (Haserick et al., 2017a; Haserick et al., 2017b). Additionally, significant differences in the glycosylation pathways were known between apicomplexans and the hosts and among various apicomplexan lineages (Liu et al., 2016; Florin-Christensen et al., 2021; Toustou et al., 2022). However, information on the glycosylation pathways in *Cryptosporidium* is fragmented in the literature. There are also variations in the annotations of certain genes in the pathways in the databases (e.g., KEGG and CryptoDB) that needs attention for the glyco-parasitology community.

In this manuscript, we analyzed the *Cryptosporidium* genomes with focus on *C. parvum* and *C. muris* that represent intestinal and gastric species, reconstructed the pathways related to glycosylation pathways including the synthesis of *N*- and *O*-linked glycans and GPI-anchors. We assembled glycosylation-related pathways to serve as a comprehensive but concise resource for the glyco-parasitology and cryptosporidium research communities. Features of glycosylation in cryptosporidium parasites and biological implications were also discussed.

## Reconstruction of the glycosylation from the *Cryptosporidium* genomes

Several approaches were employed to identify genes encoding protein glycosylation enzymes, including those for the biosynthesis nucleotide-sugars, *O*- and *N*-linked glycans and GPI-anchors. We focused on *C. parvum* and *C. muris* that represented intestinal and gastric species that show major differences in the *N*-glycoforms. All protein sequences predicted from the genomes of *C. parvum* (Iowa-II and ATCC strains) and *C. muris* (RN66 strain) were fetched from the CryptoDB (https://www.cryptodb.org/; release 5.5) and used as queries for K-number assignment using BlastKOALA tool (https://www.kegg.jp/blastkoala/) at the Kyoto Encyclopedia of Genes and Genomes (KEGG) database (Kanehisa et al., 2022). Pathways reconstructed with the assigned K numbers were compared with these already annotated by KEGG for *C. parvum*. Enzymes mapped to the glycosylation-related pathways were subjected to analysis for protein families and domains by InterProScan (https://www.ebi.ac.uk/interpro/search/sequence/) (Mitchell et al., 2019). Transmembrane domain (TMD) topology and signal peptides (SPs) were predicted using Phobius (https://phobius.sbc.su.se) (Kall et al., 2007). Some protein sequences were further analyzed by BLAST searches for information on their homologs and conserved domains against the NCBI protein databases and conserved domain databases (CDD). Potential nucleotide sugar transporters were identified by combining results from InterProScan and TransportDB (http://www.membranetransport.org/transportDB2/) (Elbourne et al., 2017), together with a recent annotation of transporters of the re-sequenced *C. parvum* genome (Baptista et al., 2022).

## *Cryptosporidium* genomes encode enzymes for synthesizing *N*-linked glycans that are much simpler than those in the hosts and differ between intestinal and gastric species

**FIGURE 1:**
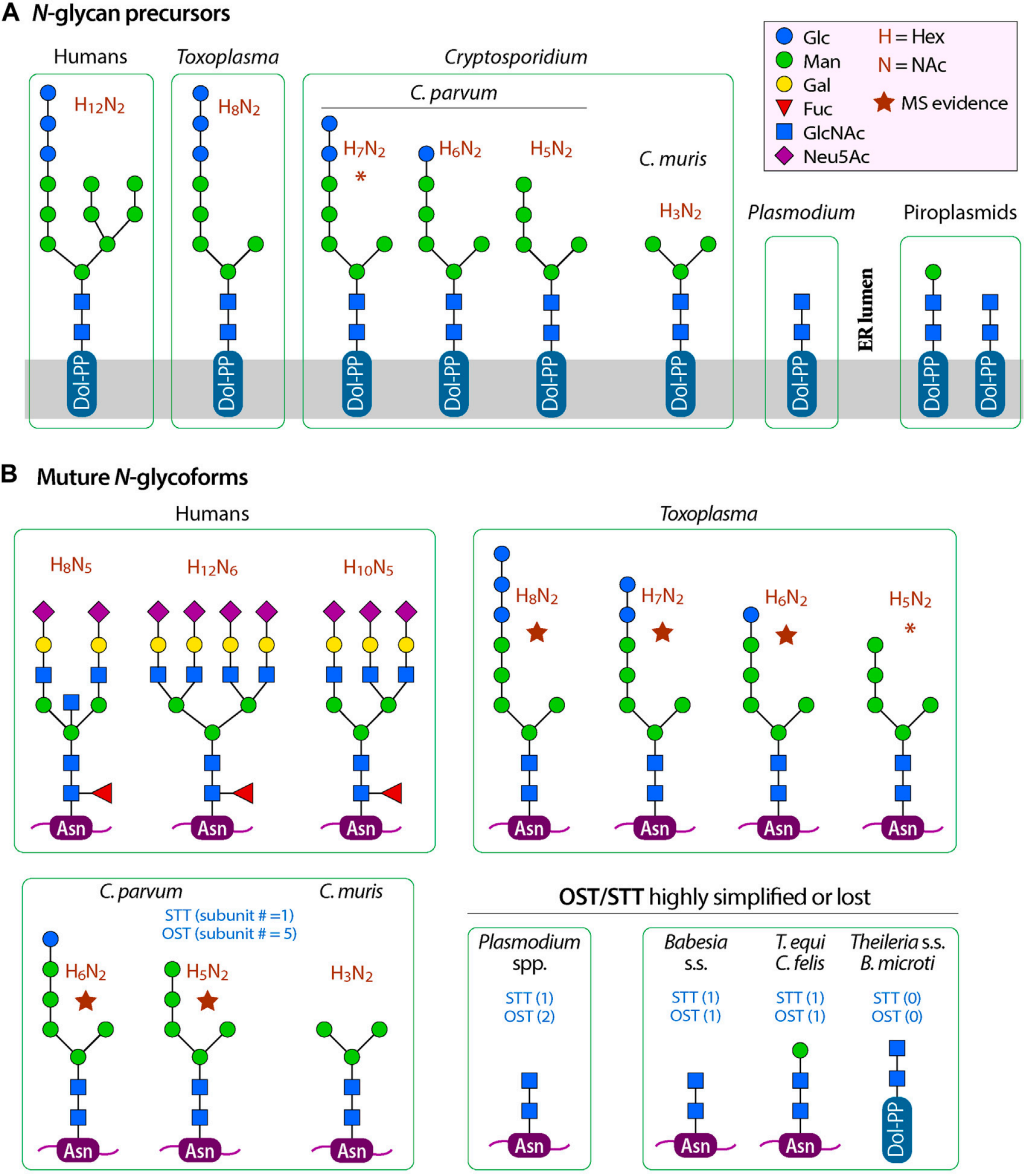
Comparison of *N*-glycan precursors **(A)** and typical mature glycoforms **(B)** between humans, *Cryptosporidium* species and other apicomplexans. Five-point purple stars indicate that the glycoforms have been detected by mass spectrometric analysis in the literature. Asterisks (*) indicate: 1) the glycan precursor H_7_N_2_ (Hex_7_NAc_2_) in *C. parvum* is undetected by mass spectrometric analysis in the literature, but the form may exist if ALG8 performs its function; or 2) the glycoform H_5_N_2_ (Hex_5_NAc_2_) in *T. gondii* is undetected by mass spectrometric analysis in the literature, but the form may exist if GANAB performs two consecutive trimming of the terminal glucoses. OST, oligosaccharyltransferase complex; STT3, oligosaccharyltransferase STT3 subunit (catalytic).

In mammals and yeasts, the *N*-glycan synthetic pathway starts with the synthesis of glycan precursor (Glc_3_Man_9_GlcNAc_2_-PP-Dol) on the endoplasmic reticulum membrane (ER), followed by the transfer of the precursor to a nascent protein at selected Asn residues (Glc_3_Man_9_GlcNAc_2_-Asn). Terminal sugars are trimmed in the ER and the Golgi into a core structure (i.e., Man_3_GlcNAc_2_) that is then extended in the Golgi to form complex glycans (Chung et al., 2017; Corfield, 2017; Toustou et al., 2022). In the synthesis of precursor, polyprenol (unsaturated long-chain isoprenoid alcohol) is first reduced by polyprenol reductase (PPR) to dolichol (Dol). Dol is phosphorylated by dolichol kinase (DolK) to form Dol-P on the cytosolic side of the ER membrane. Dol-P is extended with oligosaccharides by a set of asparagine-linked glycosylation (ALG) enzymes. These include ALG7 and ALG13/ALG14 to add two *N*-acetyl-D-glucosamines (GlcNAc_2_), and ALG1 and ALG2 to add three mannoses (Man_3_) to form the “core” structure (i.e., Man_3_GlcNAc_2_-PP-Dol). The core is extended by ALG11 by adding two more mannoses to one of the two arms. The saccharide chain on the PP-Dol is then translocated into the ER luminal side for adding four more mannoses to form three branches by ALG3, ALG9 and ALG12. Three glucoses are then added to one of the branch by ALG6, ALG8 and ALG10 to form the precursor Glc_3_Man_9_GlcNAc_2_-PP-Dol (see Figure 1A for illustration of *N*-glycan precursors in humans and apicomplexans).

**FIGURE 2:**
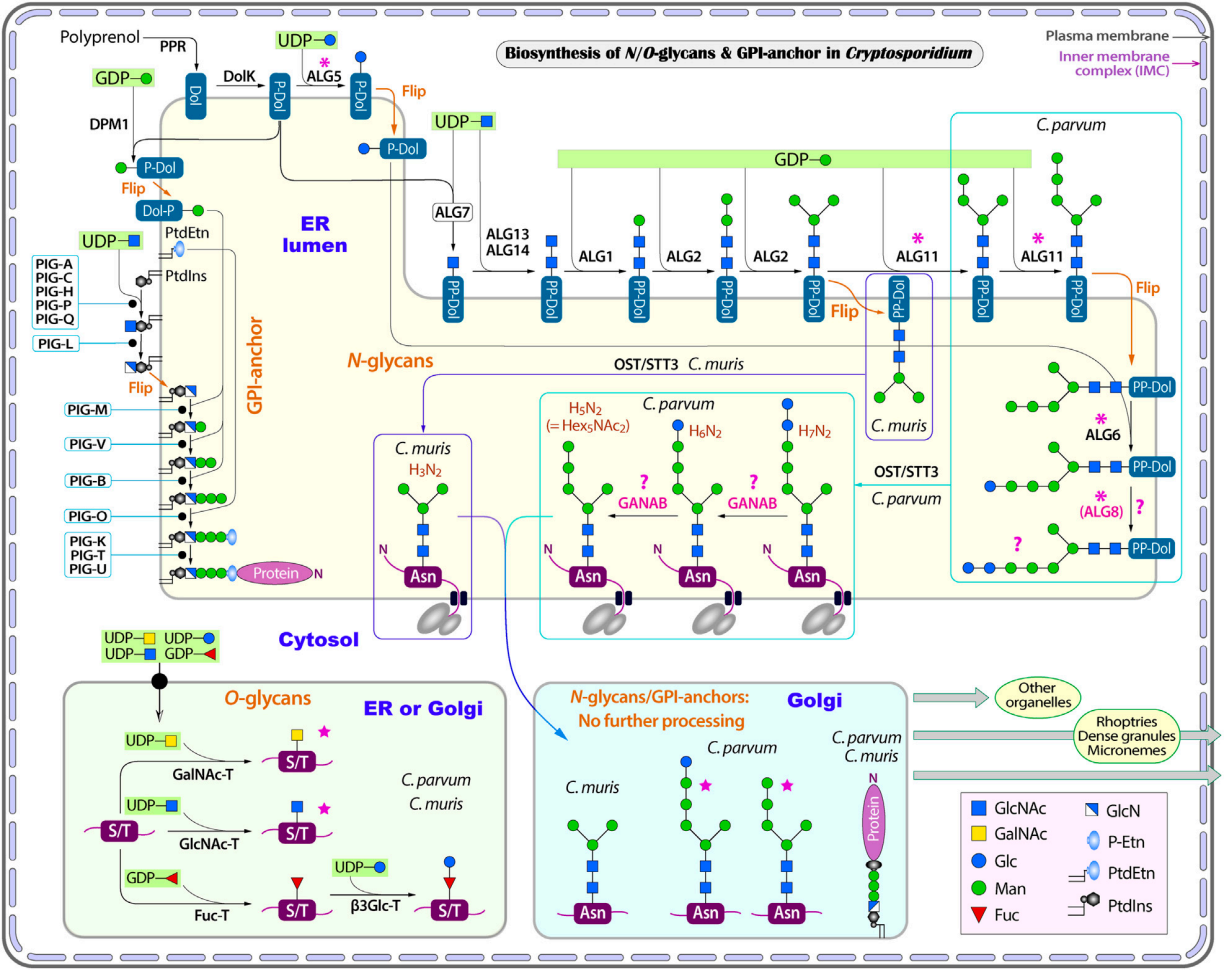
Illustration of glycosylation pathways in *Cryptosporidium* as exemplified with *C. parvum* (intestinal species) and *C. muris* (gastric species). These include the biosynthesis of *N*- and *O*-linked glycans and glycosylphosphatidylinositol (GPI) anchor in the endoplasmic reticulum (ER) and the Golgi apparatus. Five-point purple stars indicate that the glycoforms have been detected by mass spectrometric analysis in the literature. Asterisks indicate enzymes that are present in the intestinal *Cryptosporidium* species (e.g., *C. parvum*) but absent in gastric species (e.g., *C. muris*). Question marks indicate questionable enzymes or glycoforms. Enzyme abbreviations: ALG, asparagine-linked glycosylation enzyme series; β3Glc-T, UDP-glucose:*O*-linked fucose beta-1,3-glucosyltransferase; DolK, dolichol kinase; DPM1, dolichol-phosphate mannosyltransferase 1; Fuc-T, peptide-*O*-fucosyltransferase; GANAB, trimming glucosidase II (mannosyl-oligosaccharide alpha-1,3-glucosidase GANAB); GlcNAc-T, polypeptide *N*-acetylglucosaminyltransferase; GalNAc-T, polypeptide *N*-acetylgalactosaminyltransferase; OST, oligosaccharyltransferase complex; PIG, phosphatidylinositol glycan enzyme series; PPR, polyprenol reductase; STT3, oligosaccharyltransferase STT3 subunit (catalytic). Compound abbreviations: Fuc, fucose; Gal, Galactose; GalNAc, *N*-acetylgalactosamine; Glc, glucose; GlcN, glucosamine; GlcNAc, *N*-acetylglucosamine; Man, mannose; P-Etn, phosphoethanolamine; PtdEtn, phosphatidylethanolamine; PtdIns, phosphatidylinositol.

In *Cryptosporidium*, all enzyme orthologs responsible for the synthesis of the “core” could be identified from their genomes. In the reference genome based on Iowa-II strain, these enzymes and gene IDs are PPR (cgd5_1340), DolK (cgd2_1560), ALG7 (cgd5_2240), AGL13/ALG14 (cgd8_4083 and cgd7_4933), ALG1 (cgd7_1810) and ALG2 (cgd1_230) (Table 1 and Figure 2). Enzymes for the extension of the long arm, up to the addition of two mannoses and two terminal glucoses, are present in the intestinal *Cryptosporidium* species (e.g., *C. parvum* and *C. hominis*). These include ALG11 (cgd4_2990), ALG6 (cgd4_3120) and ALG8 (cgd1_2100). Therefore, the predicted *N*-glycan precursor in *C. parvum* and other intestinal species is Glc_2_Man_5_GlcNAc_2_, which is simpler than that in the hosts (Figure 1A).

**TABLE 1:** *Cryptosporidium parvum* enzymes in the biosynthesis of *N*-linked glycans.

Enzyme^a^	EC No.	Gene ID^b^	Length (aa)	SP	TMD	CryptoDB description
PPR	1.3.1.22; 1.3.1.94	cgd5_1340	273	No	7	3-oxo-5-alpha-steroid 4-dehydrogenase C-terminal domain containing protein
DolK	2.7.1.108	cgd2_1560	532	No	15	Polyprenol kinase family
ALG7	2.7.8.15	cgd5_2240	424	No	10	Glycosyl transferase family 4
ALG13	2.4.1.141	Atcc_004267/cgd8_4083	198/121	No	0	Glycosyltransferase family 28 C-terminal domain containing protein
ALG14	2.4.1.141	cgd7_4933	672	No	4	ALDH/Gamma-glutamyl phosphate reductase
ALG1	2.4.1.142	cgd7_1810	680	No	1	Chitobiosyldiphosphodolichol beta-mannosyltransferase ALG1-like
ALG2	2.4.1.132 2.4.1.257	cgd1_230	463	No	1	Glycosyl transferase, family 1
ALG11*	2.4.1.131	cgd4_2990	447	Yes	3	Glycosyl transferase
ALG6*	2.4.1.267	cgd4_3120	529	Yes	11	Glycosyl transferase ALG6/ALG8
ALG8*	2.4.1.265	Atcc_000200/cgd1_2100	505/470	No	13	Alpha-1, 3-glucosyltransferase
ALG5*	2.4.1.117	cgd5_2590	362	No	1	Glycosyltransferase 2-like
STT	2.4.99.18	cgd6_2040	722	No	13	Oligosaccharyl transferase STT3 protein
OST	2.4.99.18	cgd2_1650	468	Yes	1	Dolichyl-diphosphooligosaccharide-protein glycosyltransferase subunit
OST	2.4.99.18	cgd5_2300	132	No	3	DAD/Ost2
OST	2.4.99.18	cgd6_5070	487	Yes	1	Ribophorin I
OST	2.4.99.18	cgd7_5080	670	Yes	3	Oligosaccharyltransferase subunit Ribophorin II
OST	2.4.99.18	cgd8_5220	343	Yes	4	OST3/OST6 family, transporter family
GANAB ?	3.2.1.207	cgd3_1580	1387	No	0	Alpha glucosidase-like family 31 glycosyl hydrolases

^a^ Asterisks(*) indicate that the enzymes are present in intestinal species (e.g., *C. parvum* and *C. hominis*) but absent in gastric species (e.g., *C. muris* and *C. andersoni*). A question mark (?) indicates that the function of the enzyme (GANAB) as a trimming glucosidase II is questionable.

^b^ When there is a discrepancy in the annotations at a locus, gene IDs and product lengths from both Iowa-II (starting with cgd) and Iowa-ATCC (starting with Atcc) genomes are listed.

Abbreviations: DolK, dolichol kinase; PPR, polyprenol reductase; SP, signal peptide; TMD, number of transmembrane domain(s).

However, the three enzymes for further extension of the core (i.e., ALG11, ALG6 and ALG8), and the enzyme ALG5 for synthesizing Glc-P-Dol, are absent in *C. muris* and *C. andersoni* (Figure 2 and Table 1, marked with asterisks), suggesting that the *N*-glycan precursor is further simplified in the gastric *Cryptosporidium* species (i.e., Man_3_GlcNAc_2_). It is intriguing to see a significant structural diversity in the *N*-glycan precursors within a single genus. Structural diversity in the *N*-glycan precursors has also been found in the piroplasmids (Florin-Christensen et al., 2021). In fact, the *N-*glycosylation seems to be highly amendable for the protozoan parasites to adapt to varied parasitic lifestyles in diverse host environments (Mule et al., 2020; Toustou et al., 2022). For comparison with other apicomplexans, the *N*-glycan precursors in the cyst-forming coccidian *T. gondii* is predicted to be similar to that in *C. parvum*, but containing three terminal glucoses in the long arm (Figure 1A) (Fauquenoy et al., 2011; Haserick et al., 2017b). Overall, the predicted *N*-glycan precursors in *Cryptosporidium* parasites are much simpler than those in humans and animals, and there is a significant diversity in the precursors among different apicomplexan lineages.

It is noticed that ALG13 is missing in the current KEGG pathway maps for *C. parvum*, while our analysis showed the presence of a candidate ortholog for encoding ALG13 (cgd8_4083). This is due to the fact that KEGG pathway maps for *C. parvum* were based on an earlier version of the annotation of the parasite genome, in which the cgd8_4083 locus was not identified as protein-coding site due to the presence of introns. There are also variations in the annotations at the ALG13, ALG14 and ALG18 loci between the Iowa-II and Iowa-ATCC genomes in the CryptoDB. For ALG13, cgd8_4083 (Iowa-II) was predicted to contain two introns and encodes 121 amino acids, while CPATCC_004267 (Iowa-ATCC) has three introns and encode 198 amino acids. For ALG14, cgd7_4933 (Iowa-II) contains three introns and encodes 672 amino acids. However at the same region, the Iowa-ATCC genome is separated into two genes, i.e., CPATCC_003712 (two introns) and CPATCC_003713 (no intron) that encode 226 and 443 amino acids, respectively. All three predicted products are annotated as “ALDH/Gamma-glutamyl phosphate reductase” at the CryptoDB. At the ALG8 locus, cgd1_2100 (Iowa-II) contains two introns near the 5′-end, predicting a 470 aa product, while CPATCC_000200 contains three introns near the 5′-end and encodes a 505 aa product. Overall, variances in intron prediction is the cause of discrepancies in annotations, which could be clarified by careful sequencing the RT-PCR products derived from their transcripts.

## *Cryptosporidium* possesses a full set of oligosaccharyltransferase subunits to transfer *N*-glycan precursors to proteins, but lacks enzymes for further processing

Both gastric and intestinal *Cryptosporidium* species possess a set of genes encoding subunits of oligosaccharyltransferase (OST) complex responsible for the transfer of glycan precursors to the Asn residue of targeted proteins (Figure 2 and Table 1). These include orthologs for subunits alpha (ribophorin I; cgd6_5070), beta (Wbp1p; cgd2_1650), gamma (cgd8_5220), delta (ribophorin II; cgd7_5080), epsilon (DAD1/Ost2-like; cgd5_2300) and STT3 (cgd6_2040). The OST complex is slightly simpler than that in humans that contains two catalytic STT3 subunits (i.e., STT3A and STT3B), but more complex than those in the piroplasmids (Shrimal and Gilmore, 2019; Florin-Christensen et al., 2021).

However, *Cryptosporidium* parasites lacks enzymes to further process the *N*-glycans in the ER and Golgi, including those to trim the terminal sugars that takes place in the ER and Golgi and those to extend the core to build more complex glycans in the Golgi (Figure 2). It is noticed that an ortholog of the alpha glucosidase-like family 31 glycosyl hydrolase is present in both intestinal and gastric *Cryptosporidium* parasites (e.g., cgd5_2300 in *C. parvum* and CMU_011750 in *C. muris*). The ortholog from *C. parvum* is annotated in the KEGG as mannosyl-oligosaccharide alpha-1,3-glucosidase (GANAB; aka glucosidase 2). In humans, GANAB is responsible for trimming the second and third glucoses after the removal of the first glucose on the long arm of the glycan precursor. However, both cgd5_2300 and CMU_011750 gene products lack an N-terminal SP and transmembrane domains (TMDs), indicating that these orthologs are not targeted to the ER, thus unable to function as GANAB. Additionally, the present of orthologs in *C. muris* and *C. andersoni* also implies that these family 31 glycosyl hydrolase orthologs unlikely function as GANAB, as gastric species lack any terminal glucoses for GANAB to act on. For comparison, GANAB in humans (UniProt: Q14697) and *T. gondii* (Gene ID: TGME49_253030) contains a signal peptide for targeting to the ER. For discussion, the “GANAB” ortholog is listed in Table 1 and Figure 2, but marked with question marks.

As mentioned earlier, *Cryptosporidium* parasites lacks enzymes to further extend *N*-glycans in the Golgi. If they truly lack GANAB to trim the two terminal glucoses, the predicted final *N*-glycoform in *C. parvum* glycoproteins would contain two terminal glucoses (i.e., Glc_2_Man_5_GlcNAc_2_) (Figure 1B). However, a mass spectrometry-based analysis of *N*-glycans in glycoproteins isolated from *C. parvum* oocysts has detected only two *N*-glycoforms (Haserick et al., 2017b). The most abundant form is Hex_6_HexNAc_2_, suggesting the presence of a single terminal glucose on the long arm (i.e., Glc_1_Man_5_GlcNAc_2_). The less abundant form is Hex_5_HexNAc_2_, suggesting the absence of any glucose on the long arm (i.e., Man_5_GlcNAc_2_). The authors speculated “that ALG8, which adds the second glucose to the *N*-glycan precursor, is not active, or a glucose residue is rapidly removed by glucosidase 2 from Glc_2_Man_5_GlcNAc_2_ after it is transferred to the nascent peptide”. The ALG8 ortholog in *C. parvum* (based on CPATCC_000200 product) is predicted to be functional based on the sequence similarity with ALG6/ALG8 enzymes from other species and the presence of catalytic residue Asp in the first non-cytosolic domain between transmembrane domains 1 and 2.

Considering that the OST complexes are highly homologous within the *Cryptosporidium* genus and that in the gastric species could transfer a smaller core Man_3_GlcNAc_2_, it is possible that the OST complex in the intestinal *C. parvum* is a relatively “sloppy” enzyme, capable of transferring varied *N*-glycan precursors to proteins. Precursors might be transferred to proteins quickly after Man_5_GlcNAc_2_ is translocated from the cytosolic side of the ER membrane to the luminal face and/or after the addition of one glucose to the long arm (GlcMan_5_GlcNAc_2_) before the action of ALG8. The presence of multiple *N*-glycoforms was also observed in *T. gondii*, in which mass spectrometry analysis detected both Hex_8_NexNAc_2_ and Hex_7_NexNAc_2_ in GAP50 protein (Fauquenoy et al., 2011). The predicted precursor in *T. gondii* is Hex_9_NexNAc_2_, so that the presence of both Hex_8_NexNAc_2_ and Hex_7_NexNAc_2_ would be a result of incomplete trimming of terminal glucoses and/or the quick transfer of non-glucosylated or partially glucosylated precursors. Nonetheless, these speculations need experimental validation, such as the confirmation of the presence and subcellular localization of GANAB, ALG8 and other enzymes and their substrate specificities. Considering that the two mature glycoforms detected by Haserick and colleagues (i.e., Hex_6_HexNAc_2_ and Hex_5_HexNAc_2_) were derived from a limited number of glycoproteins from the oocysts, one could not exclude the possible presence of Hex_7_HexNAc_2_ in other glycoproteins in the oocysts or in the glycoproteins from other developmental stages of the parasite. Because of the uncertainty of the final forms of precursors, we list three hypothetic precursors in *C. parvum* (Figures 1, 2).

## *Cryptosporidium* parasites produce mainly one-unit *O*-linked glycans

In eukaryotes, *O*-glycosylation occurs in the ER and Golgi (Van den Steen et al., 1998; Joshi et al., 2018). Humans and animals make a diverse of *O*-glycans attached to the hydroxyl groups of Ser or Thr (S/T) residues in a protein, including mucin-type, mannose-type and other types of *O*-glycans. The oligosaccharides of *O*-glycans can be single-chained or branched. Both gastric and intestinal *Cryptosporidium* species contain three types of *O*-glycan transferases (Figure 2 and Table 2): protein *O*-fucosyltransferase (Fuc-T; cgd1_2440), protein *O*-GlcNAc transferase (GlcNAc-T; cgd1_1300) and five candidates for polypeptide GalNAc transferase (GalNAc-T; cgd5_690, cgd6_1960, cgd7_1310, cgd7_4120 and cgd7_4160). These transferases possess either a signal peptide or one to two TMDs for targeting to the ER/Golgi where *O*-glycosylation occurs (Table 2). *Cryptosporidium* lacks enzymes to add more sugars to the one-unit *O*-GlcNAc and *O*-GalNAc glycans, but possesses a putative UDP-glucose:*O*-linked fucose beta-1,3-glucosyltransferase (β3Glc-T; cgd5_540) to potentially extend the *O*-Fuc with a glucose. Therefore, *Cryptosporidium* parasites are predicted to make only three types of simple *O*-glycoforms: GlcNAc(β1)-S/T, GalNAc(β1)-S/T and Glc(β1-3)Fuc(α3)-S/T (Figure 2).

**TABLE 2:** *Cryptosporidium parvum* enzymes in the biosynthesis of *O*-linked glycans.

Enzyme	EC No.	Gene ID*	Length (aa)	SP	TMD	CryptoDB description
Fuc-T	2.4.1.221	cgd1_2440	461	Yes	0	Uncharacterized signal peptide-containing protein
β3Glc-T	2.4.1.-	Atcc_002050/cgd5_540	574/196	No	1	unspecified product/Uncharacterized protein
GlcNAc-T	2.4.1.255	cgd1_1300	1032	No	1	Spindly like TPR repeat containing protein
GalNAc-T	2.4.1.41	cgd5_690	732	No	1	Glycosyl transferase family 2
GalNAc-T	2.4.1.41	cgd6_1960	809	No	3	Glycosyltransferase 2-like
GalNAc-T	2.4.1.41	cgd7_1310	669	No	2	Glycosyltransferase 2-like protein
GalNAc-T	2.4.1.41	cgd7_4120	414	Yes	0	Glycosyltransferase 2-like
GalNAc-T	2.4.1.41	cgd7_4160	637	No	1	Glycosyltransferase 2-like

*When there is a discrepancy in the annotations at a locus, gene IDs and product lengths from both Iowa-II (starting with cgd) and Iowa-ATCC (starting with Atcc) genomes are listed.

Abbreviations: SP, signal peptide; TMD, number of transmembrane domain(s).

The annotation of cgd5_540 and orthologs are less certain, though. At this locus, the earliest *C. parvum* (Iowa-II) genome project predicted a 507-aa long partial “hypothetical protein” that contains a domain homologous to galactosyltransferase (GenBank: XP_001388252; two introns). The current annotation of the Iowa-II genome in the CryptoDB predicted a 196 aa protein (no introns). The annotation of the Iowa-ATCC genome predicted (CPATCC_002050; four introns) a 574 aa “unspecified product”. The CPATCC_002050 product is the longest of the three predictions, containing a Fringe domain at the C-terminal half of the protein (InterProScan ID: IPR017374). Fringe proteins are a family of glycosyltransferases, including beta-1,3-glucosyltransferase (i.e., β3Glc-T) that glucosylates *O*-linked fucosylglycan on thrombospondin type 1 repeat domains in humans (Sato et al., 2006). The *C. parvum* ortholog contains no signal peptide, but has a TMD very close to the N-terminus (amino acid positions from 6 to 22). The architecture resembles human β3Glc-T (UniProt: Q6Y288; GenBank: NP_919299), but the long domain is predicted to be cytosolic, rather than non-cytoplasmic by Phobius and InterProScan. Collectively, it is certain that the cdg5_540 locus encodes a glycosyltransferase, likely β3Glc-T, but its true identity and function need experimental validation.

*O*-glycans in proteins from the *C. parvum* oocysts were detected by mass spectrometry-based analysis that identified HexNAc from four glycoprotein (i.e., gp40, gp15, gp20 and gp900) (Haserick et al., 2017a). The acetylhexosamine was thought to be likely GalNAc by the authors based on earlier report on the presence of four GalNAc-Ts. The presence of a putative GlcNAc-T in the *C. parvum* genome suggests that both GalNAc and GlcNAc might exit. The same study failed to detect Glc-Fuc or Fuc, which might be due to the fact that only four highly abundant glycoproteins from a single developmental stage (oocysts) were analyzed. Further experiments are needed to validate the presence/absence and the scale of *O-*fucosylation in the parasite.

## *Cryptosporidium* parasites synthesize only three nucleotide-sugars *de novo* as glycan substrates, but possess at least 12 transporters with substrate preference towards nucleotide sugars or phosphorylated sugars

**FIGURE 3:**
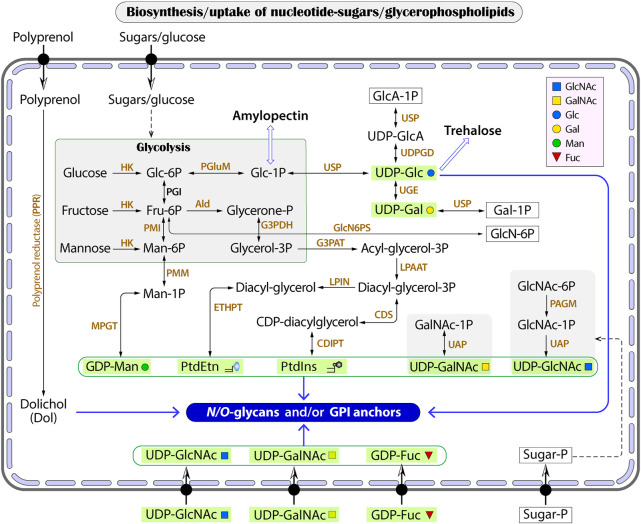
The biosynthesis of glycan substrates derived from glycolysis or conversions between phosphorylated HexNAc (GalNAc-1P and GlcNAc-1P) and UDP-HexNAc. Transporters for nucleotide sugars and phosphorylated sugars are illustrated without naming of specific proteins. Wide arrows indicate multi-step reactions. Enzyme abbreviations: Ald, aldolase; CDIPT, CDP-diacylglycerol-inositol 3-phosphatidyltransferase (phosphatidylinositol synthase); CDS, CDP-diacylglycerol synthase; ETHPT, ethanolaminephosphotransferase; G3PAT, glycerol-3-phosphate acyltransferase; G3PDH, glycerol-3-phosphate dehydrogenase; HK, hexokinase; LPIN, phosphatidate phosphatase LPIN; LPAAT, lysophosphatidic acid-acyltransferase (LPA acyltransferase); PAGM, phosphoacetylglucosamine mutase; PGI, phosphoglucose isomerase; PGluM, phosphoglucose mutase; PMGD, mannose-1P guanylyltransferase; PMI, phosphomannose isomerase; PMM, phosphomannose mutase; UAP, UDP-*N*-acetylglucosamine/UDP-*N-*acetylgalactosamine diphosphorylase; UDPDG, UDP-glucose 6-dehydrogenase; UGE, UDP-glucose 4-epimerase; USP, UDP-sugar pyrophosphorylase (UDP-*N*-acetylglucosamine pyrophosphorylase). Compound abbreviations: Fru, fructose; Gal, galactose; GalNAc, *N*-acetylgalactosamine; Glc, glucose; GlcA, glucuronic acid; GlcN, glucosamine; GlcNAc, *N*-acetylglucosamine; Man, mannose; PtdEtn, phosphatidylethanolamine; PtdIns, phosphatidylinositol.

While humans and animals synthesize all nucleotide sugar substrates for use in glycosylation, *Cryptosporidium* only makes GDP-Man, UDP-Glc and UDP-Gal from the glycolytic pathway (Figure 3 and Table 3). The parasite lacks enzymes to make other nucleotide sugars *de novo*, including GDP-Fuc, UDP-GlcNAc and UDP-GalNAc. It contains an ortholog of dual functional UDP-*N*-acetylglucosamine/UDP-N-acetylgalactosamine diphosphorylase (UAP; cgd4_810) that may potentially convert phosphorylated N-acetylated amino sugars (e.g., GlcNAc-1P and GalNAc-1P) into UDP-sugars (e.g., UDP-GlcNAc and UDP-GalNAc). In *Cryptosporidium*, GDP-Man is used in synthesizing both *N*-glycans and GPI-anchor (on the cytosolic and luminal side of the ER, respectively) (Figure 3), while other nucleotide sugars are used in synthesizing *N*- and *O*-glycans. *Cryptosporidium* lacks enzymes to make GDP-Fuc from either fucose or GDP-Man (vs. humans possessing both pathways), so that the parasite has to scavenge GDP-Fuc from the host for use in *O*-fucosylation. It is worth to mention that *Cryptosporidium* is unable to synthesize nucleosides and pentoses *de novo*, but contains enzymes to produce various nucleotides from nucleosides (e.g., GDP and UDP) (Abrahamsen et al., 2004; Xu et al., 2004; Rider and Zhu, 2010).

**TABLE 3:** *Cryptosporidium parvum* enzymes in the *de novo* synthesis of nucleotide sugars.

Enzyme	EC No.	Gene ID	Length (aa)	SP	TMD	CryptoDB description
PMM	5.4.2.8	cgd4_960	242	No	0	Phosphomannomutase
MPGT	2.7.7.13	cgd2_1770	425	No	0	Mannose-1-phosphate guanylyltransferase
USP	2.7.7.64	cgd7_1830	654	No	1	UTP-glucose-1-phosphate uridylyltransferase
UGE	5.1.3.2	cgd4_2600	345	No	0	UDP-glucose 4-epimerase
AGM	5.4.2.3	cgd4_3310	643	No	0	Phosphoacetylglucosamine mutase
UAP	2.7.7.23; 2.7.7.83	cgd4_810	603	No	0	UDP-N-acetylglucosamine pyrophosphorylase

Abbreviations: SP, signal peptide; TMD, number of transmembrane domain(s).

On the other hand, *Cryptosporidium* possesses at least 12 transporters with predicted substrate specificity on nucleotide-sugars based on genome analysis with InterProScan and TransportDB, together with the annotation of transporters on the Iowa-ATCC genome of *C. parvum* by other investigators (Table 4). These include six putative GDP-Fuc transporters, in which three are arranged in tandem in chromosome 3 (i.e., cgd3_490, cgd3_500 and cgd3_510). There are four UDP-sugar transporters for UDP-GlcNAc or UDP-Gal, in which two are arranged in tandem in chromosome 2 (i.e., cgd2_2660 and cgd2_2670). There are two putative transporters for phosphorylated sugars. There is a need to determine the subcellular localizations of these transporters to confirm whether they are responsible for scavenging substrates from the host and/or for transporting substrates into the ER/Golgi lumens. The true substrates of these putative transporters also remain to be elucidated.

**TABLE 4:** Transporters with predicted substrates on nucleotide sugars in *Cryptosporidium parvum*.

Gene ID	Length (aa)	TMD	Putative substrates	CryptoDB description	TransportDB prediction	InterPro description
cgd3_490	432	8	GDP-Fuc	GDP-fucose transporter	GDP-fucose	Sugar phosphate transporter domain; GDP-fucose transporter
cgd3_500	394	10	GDP-Fuc	GDP-fucose transporter	GDP-fucose	Sugar phosphate transporter domain; GDP-fucose transporter
cgd3_510	389	8	GDP-Fuc	GDP-fucose transporter	GDP-fucose	Sugar phosphate transporter domain
cgd4_3110	736	10	GDP-Fuc/Sugar-P	Sugar phosphate transporter domain containing protein	GDP-fucose	Sugar phosphate transporter domain; GDP-fucose transporter
cgd8_1370	428	10	GDP-Fuc/Sugar-P	GDP-fucose transporter with 9 transmembrane domains	GDP-fucose	Sugar phosphate transporter domain; GDP-fucose transporter
cgd8_1440	417	10	Sugar-P/GDP-Fuc	Triose-phosphate Transporter	GDP-fucose	Sugar phosphate transporter domain; GDP-fucose transporter
cgd1_40	337	9	UDP-Gal	UDP-galactose transporter/UAA transporter	UDP-galactose	UAA transporter
cgd2_2660	430	10	UDP-GlcNAc	UDP N-acetylglucosamine transporter-like nucleotide sugar transporter	UDP-N-acetylglucosamine	Nucleotide-sugar transporter
cgd2_2670	414	10	UDP-GlcNAc	UDP N-acetylglucosamine transporter-like nucleotide sugar transporter	UDP-N-acetylglucosamine	Nucleotide-sugar transporter
cgd2_590	453	9	UDP-GlcNAc	Nucleotide-sugar transporter	UDP-N-acetylglucosamine	Nucleotide-sugar transporter
cgd5_3410	495	10	Sugar-P	Triose-phosphate transporter family	phosphate/phosphoenolpyruvate	Sugar phosphate transporter domain
cgd8_1600	789	12	Sugar-P	Sugar phosphate permease	sugar phosphate	Major facilitator superfamily; MFS transporter superfamily

TMD, number of transmembrane domains.

The *Cryptosporidium* genomes also encode a “mannose-P-dolichol utilization defect 1” (MPDU1; cgd2_550) that was found to be required for Dol-P-monosaccharide-dependent glycosyltransferase activities in mammals (Anand et al., 2001; van Tol et al., 2019). In *Cryptosporidium*, MPDU1 would play roles in assisting the synthesis of *N*-glycans and GPI-anchors that utilize Dol-P-Glc and Dol-P-Man as substrates, respectively.

## *Cryptosporidium* parasites synthesize unbranched GPI-anchors

The GPI-anchor in *Cryptosporidium* is predicted to be unbranched. Both gastric and intestinal *Cryptosporidium* species contain a set of genes encoding various classes of phosphatidylinositol glycan (PIG) protein (Table 5 and Figure 2). The GPI-anchor biosynthesis starts with the action of phosphatidylinositol *N*-acetylglucosaminyltransferase complex that catalyzes the attachment of GlcNAc to the phosphatidylinositol (PtdIns) on the cytosolic side of the ER membrane (Kinoshita and Fujita, 2016; Morotti et al., 2019). This complex in *Cryptosporidium* consists of five subunits, including PIG-A (cgd4_2100), PIG-C (cgd1_3380), PIG-H (cgd6_4600), PIG-P (cgd2_840) and PIG-Q (cgd5_1850). The parasite contains PIG-L (cgd7_700) to deacetylate the attached GlcNAc to GlcN (glucosamine), also on the cytosolic side of the ER membrane. PtdIns-GlcN is translocated into the ER luminal side, from which three mannoses (Man1, Man2 and Man3) from Dol-P-Man are sequentially attached to GlcN by PIG-M (cgd1_3720), PIG-V (cgd6_5100) and PIG-B (cgd3_3590), respectively. Dol-P-Man is synthesized by Dol-P-Man synthase (DPM1; cgd5_2040). In humans and animals, Dol-P-Man is also a donor of mannoses in synthesizing complex *N*-glycans (e.g., high-mannose *N*-glycans) that are absent in *Cryptosporidium*. Therefore, Dol-P-Man seems to be solely used as a substrate in GPI-anchor synthesis in the parasite.

**TABLE 5:** *Cryptosporidium parvum* enzymes in the biosynthesis of GPI-anchor*.

Enzyme	EC No.	Gene ID	Length (aa)	SP	TMD	CryptoDB description
DPM1	2.4.1.83	cgd5_2040	242	No	0	Glycosyl transferase family 2
PIG-A	2.4.1.198	cgd4_2100	444	0	1	Glycosyl transferase family 1/GPI anchor biosynthesis related protein
PIG-C	2.4.1.198	cgd1_3380	274	0	8	Phosphatidylinositol N-acetylglucosaminyltransferase subunit C
PIG-H	2.4.1.198	cgd6_4600	182	0	2	GPI-GlcNAc transferase complex/PIG-H component
PIG-P	2.4.1.198	cgd2_840	206	0	2	PIG-P
PIG-Q	2.4.1.198	cgd5_1850	670	0	6	GPI1/PIG-Q like N-acetylglucosaminyl-phosphatidylinositol transferase involved in GIP anchor biosynthesis
PIG-L	3.5.1.89	Atcc_003223/cgd7_700	220/175	0	1	N-acetylglucosaminyl-phosphatidylinositol de-N-acetylase
PIG-M	2.4.1.-	Atcc_000018/cgd1_3720	462/382	0	8	Mannosyltransferase DXD
PIG-V	2.4.1.-	cgd6_5100	440	0	9	GPI mannosyltransferase 2
PIG-B	2.4.1.-	cgd3_3590	625	0	9	GPI mannosyltransferase
PIG-O	2.7.-.-	cgd8_2260	1041	0	13	Alkaline-phosphatase-like protein
PIG-K		cgd1_220	426	0	2	Glycosylphosphatidylinositol transamidase
PIG-T		cgd6_3240	585	SP	2	Gpi16p/PIG-T/SPBC1604.15 family glycosyl phosphatidyl inositol
PIG-U		cgd7_540	466	0	9	GPI transamidase subunit PIG-U

*When there is a discrepancy in the annotations at a locus, gene IDs and product lengths from both Iowa-II (starting with cgd) and Iowa-ATCC (starting with Atcc) genomes are listed.

SP, signal peptide; TMD, number of transmembrane domain(s).

In the final two steps, a “bridging” ethanolamine-phosphate (Etn-P) is transferred from phosphatidylethanolamine (PtdEtn) to the 6-position of Man3 by PIG-O (cgd8_2260) (Figure 2). PtdEtn can be derived from the glycolytic pathway in *Cryptosporidium* that contains a full set of five enzymes to convert glycerone-P to PtdEtn. These include glycerol-3-phosphate dehydrogenase (G3PDH; cgd2_210), glycerol-3-phosphate acyltransferase (G3PAT; cgd6_1270), two lysophosphatidic acid-acyltransferases (LPAAT1; cgd2_4050 and LPAAT2; cgd8_1400), phosphatidate phosphatase (LPIN; cgd3_3210), and ethanolamine-phosphotransferase (ETHPT; cgd4_2790). Finally, GPI transamidase consisting of three subunits, i.e., PIG-K (cgd1_220), PIG-T (cgd6_3240) and PIG-U (cgd7_540), catalyzes the attachment of GPI to a targeted protein. In this reaction, PIG-K acts as a caspase-like cysteine peptidase that cleaves off the C-terminal GPI attachment signal peptide.

There are some discrepancies in the annotation of three genes between the two *C. parvum* genomes. 1) DPM1 gene in the Iowa-II genome (cgd5_2040) contains four introns and encodes a 242 aa product, while that in Iowa-ATCC (CPATCC_002211) contains four introns and encodes a 219 aa product. The intron predictions are identical in both annotations, but the open reading frame of cgd5_2040 contains 69 extra coding sequences in the upstream of the gene. 2) PIG-L in Iowa-II (cgd7_700) contains two introns and encodes a 175 aa, while in Iowa-ATCC (CPATCC_003223) it contains four introns and encodes a 220 aa product. 3) PIG-M in Iowa-II (cgd1_3720) is predicted to have one intron and encode a 382 aa product, while that in Iowa-ATCC (CPATCC_000018) contains two introns and encodes a 462 aa product.

In comparison to humans and animals whose GPI-anchors are branched with two individual Etn-P molecules attached to Man1 (2-position) and Man3 (6-position), *Cryptosporidium* lacks PIG-N and PIG-G to add any Etn-P to the side of the GPI-anchor. *Cryptosporidium* also lacks PIG-W to attach a long fatty acyl chain to PtdIns that occurs in the humans immediately after the translocation of PtdIns-GlcN to the luminal face of ER. The GPI-anchor in *Cryptosporidium* is not processed further in the Golgi by adding additional sugars (e.g., GalNAc, Gal or sialic acid). Therefore, the final form of the GPI in the parasite is simple and unbranched, differing significantly from the hosts.

## Implication on the parasite biology

The importance of glycoproteins in *Cryptosporidium* has been indicated by their presence in all stages of the parasite, including the oocyst walls (e.g., the cryptosporidial oocyst wall proteins (COWPs) and molecules tethering sporozoites on the inner side of the oocyst wall), the surface of sporozoites (e.g., gp40/gp15, CpMuc4) and the PVM (recognizable by certain lectins such as VVL) (Petersen et al., 1992; Winter et al., 2000; Priest et al., 2001; Wanyiri and Ward, 2006; Paluszynski et al., 2014; Haserick et al., 2017a; Haserick et al., 2017b; Tandel et al., 2019; Li et al., 2022). Structural glycoproteins play protective and lubricative roles, such as those in the oocyst walls and those on the plasma membranes of zoites and PVM. These structures protect the parasite from hazardous factors in the environments or the gastrointestinal tracts. Glycoproteins distributed on the surface of zoites or discharged from the zoites also interact with host cells during the parasite invasion of host cells.

While the *N-*glycoform in the intestinal *Cryptosporidium* is of simplicity, that in the gastric species is further simplified to contain only the core structure. The further reduction of complexity of the *N-*glycoform in species of the same genus is an intriguing subject of study in the evolution of glycosylation pathways and the adaptation of parasitic lifestyles from the intestine to the stomach. The lack of enzymes to further process *N*-glycans in the Golgi is in fact a general feature in the apicomplexans based on the KEGG annotations and mass-spectrometric evidence for *Cryptosporidium, Toxoplasma* and *Plasmodium* (Priest et al., 2003; Fauquenoy et al., 2011; Haserick et al., 2017a; Haserick et al., 2017b). Hematozoa (e.g., *Plasmodium, Babesia* and *Theileria*) even make highly truncated *N*-glycans (i.e., NAc2 or Man_1_NAc_2_), in which most *Theileria* species and *B. microti* also lost all OST/STT subunits (Figure 3) (Morotti et al., 2019; Florin-Christensen et al., 2021). On the other hand, other alveolates (e.g., ciliates and dinoflagellates) may possess some enzymes to process *N*-glycans in the Golgi (e.g., various types of mannosidases and GlcNAc transferases), although not as extensive as those in vertebrates and other higher eukaryotes, based on KEGG pathway annotations and literature (Tivey et al., 2020; Toustou et al., 2022). These imply that the apicomplexan ancestor might process glycans in the Golgi, at least to a certain degree, but the ability was lost in adaptation of the intracellular parasitic lifestyle. Because apicomplexans possess an apicoplast derived from an algae *via* a secondary endosymbiosis (although it has been lost in the *Cryptosporidium* lineage), there was a hypothesis for “Darwinian selection against *N*-glycans in nucleus-encoded proteins that must pass through the ER prior to threading into the apicoplast” (Bushkin et al., 2010). The selection against glycosylation would affect non-apicoplast proteins as well (e.g., secretory proteins) because the process would apply to both apicoplast and non-apicoplast proteins in the ER or Golgi.

Another interesting feature is the presence of malectin in *Cryptosporidium* (cgd6_110 and orthologs), but absent in other apicomplexan lineages (Wu et al., 2022). Malectin in humans and animals binds to Glc_2_Man_9_GlcNAc_2_ *N*-glycan after the trimming of the terminal glucose from Glc_3_Man_9_GlcNAc_2_ to facilitate the recruitment of glucosidase II (Schallus et al., 2008; Schallus et al., 2010; Galli et al., 2011; Takeda et al., 2014). However, gastric *Cryptosporidium* lacks any terminal glucoses or mannoses but possesses malectin, indicating that *Cryptosporidium* malectin plays a function that differs from humans and animals. This notion is supported by a recent study that showed difference in binding properties between human and *Cryptosporidium* malectins (Wu et al., 2022), although the exact binding partner remains to be elucidated.

The physiochemical properties of glycans differ between *Cryptosporidium* and hosts due to the differences in their compositions and structures. These need to be considered in studying the roles of the parasite glycoproteins. The host gastrointestinal tracts are covered by a layer of mucosa consisting of a network of mucins (mainly mucin-2) that is negatively charged due to the presence of sialic acid (Boegh and Nielsen, 2015; Wagner et al., 2018). The negative charge allows the mucins to bind to positively charged molecules on microbes, thus restricting the movement of microbes. This is one of the mechanisms of protection from free access of pathogens to the epithelial cells. On the other hand, the glycans in *Cryptosporidium* are not only simplified, but also neutrally charged by lacking sialic acid. Therefore, the glycans themselves would make no or little contribution to the adhesion of the parasite glycoproteins to the host cell extracellular structures, which has been proposed for the gp900 (Li et al., 2022). Instead, glycans in the parasite mainly play a lubrication role and/or are responsible for binding to certain lectins (if any) on the host cells.

The genetically trackable *T. gondii* has been used as a surrogate system to study *C. parvum* glycoproteins (e.g., CpGP40/15) (O'Connor et al., 2003). Knowing the difference between the native glycoforms of *C. parvum* and *T. gondii* would allow investigators to consider manipulate the pathway in *T. gondii* to make true cryptosporidium-type glycans. Many vaccine candidates are also glycoproteins (Mead, 2014), in which glycans are a source of antigenicity in triggering immunological responses in the hosts. However, recombinant proteins expressed in prokaryotes are not glycosylated. Eukaryotic expression systems (e.g., yeasts, insect cell-based baculovirus or mammalian cells) might glycosylate heterogeneous proteins, but in different glycoforms. Knowing the glycoforms in specified vaccine candidates for cryptosporidiosis would guide investigators in protein glycoengineering or manipulating the glycosylation pathway in prokaryotic or eukaryotic expression systems to produce immunogens with correct glycoforms (Anyaogu and Mortensen, 2015; Hamilton and Zha, 2015; Naegeli and Aebi, 2015; Ma et al., 2020; Schon et al., 2021).
